# Specialisation versus special interest - the Australian podiatry experience

**DOI:** 10.1186/s13047-015-0127-0

**Published:** 2015-12-03

**Authors:** Ainslie Davies, Paul Bennett, Susan Nancarrow, Antonio Cuesta-Vargas

**Affiliations:** School of Clinical Sciences, Queensland University of Technology, Brisbane, Queensland Australia; Southern Cross University, Lismore, New South Wales Australia; University of Malaga, Malaga, Spain

**Keywords:** Podiatry, Health workforce, Scope of practice, Diversification, Specialisation, Substitution

## Abstract

**Background:**

Ensuring efficient and effective delivery of health care to an ageing population has been a major driver for a review of the health workforce in Australia. As part of this process a National Registration and Accreditation Scheme (NRAS) has evolved with one goal being to improve workforce flexibility within a nationally consistent model of governance. In addition to increased flexibility, there have been discussions about maintaining standards and the role of specialisation. This study aims to explore the association between practitioners’ self-perceptions about their special interest in musculoskeletal, diabetes related and podopaediatric foot care and the actual podiatry services they deliver in Australia.

**Methods:**

A cross sectional on-line survey was administered on behalf of the Australasian Podiatry Council and its’ state based member associations. Self-reported data were collected over a 3-week interval and captured information about the practitioners by gender, years of clinical experience, area of work by state, work setting, and location. For those participants that identified with an area of special interest or specialty, further questions were asked regarding support for the area of special interest through education, and activities performed in treating patients in the week prior to survey completion. Queensland University of Technology Human Research Ethics approval was sought and confirmed exemption from review.

**Results:**

218 podiatrists participated in the survey. Participants were predominately female and worked in private practices.

The largest area of personal interest by the podiatrists was related to the field of musculoskeletal podiatry (*n* = 65), followed closely by diabetes foot care (*n* = 61), and a third area identified was in the management of podopaediatric conditions (*n* = 26).

**Conclusions:**

Health workforce reform in Australia is in part being managed by the federal government with a goal to meet the health care needs of Australians into the future. The recognition of a specialty registration of podiatric surgery and endorsement for scheduled medicines was established with this workforce reform in mind. Addition of new subspecialties may be indicated based on professional development, to maintain high standards and meet community expectations.

**Electronic supplementary material:**

The online version of this article (doi:10.1186/s13047-015-0127-0) contains supplementary material, which is available to authorized users.

## Background

The efficiency and effectiveness of Australia’s health workforce has been under increasing scrutiny by the Australian Federal Governments’ Department of Health and Ageing in responses to Australia’s changing population health needs. An ageing population combined with the associated burden of chronic disease has given impetus to a growing national health workforce reform agenda. The 2005 Productivity Commission Report on Australia’s Health workforce [1] was a major catalyst for a change in thinking about innovative ways to address this chronic burden of disease. The report recommended addressing; boundaries and arrangements that limit productivity of the health workforce, lack of the health care systems’ ability to respond to communities changing needs and workforce shortages in some professions in rural and remote areas. This changing need is further evidenced by the National Health and Medical Research Council’s strategic plan 2013-2015 [2], which identifies, as a priority action, health care interventions that improve the care of patients with multiple complex chronic diseases such as diabetes and musculoskeletal disorders. With respect to the organisation and delivery of health care services, it has been noted problems exist with health care fragmentation, poor co-ordination of services, regulatory inflexibility and distortions in funding arrangements [3].

To address these issues, the Australian Health Workforce Institute (AHWI) and the Health Workforce Australia (HWA) were also established by the Council of Australian Governments (COAG) to help build a sustainable health workforce for Australia. Consequently the National Registration and Accreditation Scheme (NRAS) was implemented in 2010 to foster a competent and flexible health workforce that meets the current and future needs of the Australian community.

This paper offers the opportunity to better understand the impact these reforms are having on health professions, in particular podiatry. The purpose of this paper is to provide additional information on what scope of practice activities are undertaken in contemporary podiatry practice in Australia. This study also aims to explore the association between practitioners’ self-perceptions about their special interest in musculoskeletal, diabetes related and podopaediatric foot care and the actual contemporary scope of podiatry practice in Australia.

Historically, the scope of podiatry practice in Australia was restricted to: “The diagnosis and treatment of ailments and abnormal conditions of the human foot using medical, surgical, mechanical and electrical therapies” [4].

The prevailing philosophy from COAG has been for a more unified national approach to ensuring high standards associated with the delivery of registered health care services, while being flexible enough to meet a changing community need and addressing niche markets.

In the interests of maintaining practitioners working to full scope of podiatry practice, NRAS and the United Kingdom (U.K) regulatory authority, the Health and Care Professions Council (HCPC) have adopted ‘protected title’ only models of governance. Any functional closure by ‘scope of practice definition’ would need to be prefaced on evidence that clinical outcomes were demonstrably better.

In addition to the Health Practitioner Regulation National Law, NRAS made provision for the creation of an independent national accreditation authority, the Australian and New Zealand Podiatry Accreditation Council (ANZPAC). ANZPAC’s function is to assess educational programs of study in podiatry and provide an assurance those programs of study comply with standards set by the PBA.

The forces for change in professional boundaries are often driven by factors external to a profession. Nancarrow [5] described many of these forces from a health consumer perspective: changing societal expectations and beliefs, unmet demand and consumer preferences, rather than managerial changes: management philosophies and distribution of resources. This is supported by Borthwick [6], who listed marketisation as well as managerialism as drivers of change. An example of change through consumer preferences includes the incorporation of podiatric surgeon specialisation through general practitioner fund holding when fund managers were given a choice of service provider.

There is limited literature on scope of podiatric practice. One study on consumers of podiatry, examined types of patients that presented to a podiatry teaching clinic [7]. The papers concluded podiatrists see a diverse range of foot pathology across the life span of patients, and whilst skin lesions are highly prevalent there appeared to be fewer young people seeking professional podiatry care. Two articles in the British Journal of Podiatry which studied of scope of practice of podiatrists [8] and [9], identified the core work of a podiatrist involved: nail care, removal of corns and calluses and provision of footwear and foot-care advice. Differences were found between the scope of practice for those working in the National Health Service (NHS) and podiatrists in private practice. Private practice podiatrists were more likely to be providing nail care, and less likely to be providing footwear advice and nail surgery. These differences in part were explained by the employment of foot care assistants in the NHS, and policies of not providing nail care to “low risk” clients.

Working to “full scope of practice” has been added to the taxonomy of allied health models of care in a recent report for Health Workforce Australia [10]. The practice of working to full scope ranks above support roles, such as the delegated activities of an allied health assistant but below advanced practice (specialist) allied health practitioners. Working within a practitioners’ full scope of practice doesn’t require additional training, or changes to legislation.

Conversely, extended scope of practice maybe defined as: “A discrete knowledge and skill base *additional* to the recognised scope of practice of a profession and/or regulatory context of a particular jurisdiction. Over time extended scope of practice may become part a professions’ full scope of practice” [10]. An example of podiatric extended scope of practice is endorsement for prescribing of scheduled medicines.

To further explain the relationship between scope of practice and the concept of professional boundaries, Nancarrow and Borthwick [5] in their analysis of changing professional boundaries in the health professions undertook to divide the roles of health professionals into four main areas: Diversification (scope), specialisation, horizontal substitution and vertical substitution. Diversification and specialisation relate to the expansion of professional activities, usually within a discipline, e.g. recognition of surgical podiatry as a specialisation or, endorsements for prescribing of scheduled medicine for diversification. Horizontal substitution occurs when providers with a similar level of training and expertise, but different backgrounds undertake roles normally the domain of another discipline. An example of this would be: physiotherapists, chiropractors and podiatrists all prescribe foot orthotics, which was principally a podiatry domain. Vertical substitution describes the transfer of tasks to professional groups at different levels, e.g. podiatry assistants. From a practical perspective these definitions prove quite useful in understanding the interrelated roles and scopes of practice of various health professional groups. Podiatry has therefore been affected by the flexibility of professional boundaries. The likely impact being change in perceptions of status as key tasks are either delegated or enhanced through changed scope of practice.

## Methods

### Secondary data analysis

This paper is based on secondary data analysis from a report to the Australasian Podiatry Council taken from published survey results of member podiatrists. Queensland University of Technology Human Research Ethics approval was sought and confirmed exemption from review, exemption number 1400000791.

The design was a cross-sectional study that involved the analysis of data collected from Australian podiatrists, who represented members of the Australian Podiatry Associations. Distribution of electronic surveys occurred via the state member associations of the Australasian Podiatry Council (APodC). Responses were received from five of the six member states, excluding Western Australia. An on-line survey tool ‘Survey Monkey’® was used for data collection Additional file 1.

The survey obtained data with respect to the individuals; demographics, years of clinical experience, work setting (public or private sector) and work environment (solo practitioner, multi-podiatrist work team or inter-professional work team). Questions were asked of self-perception of work practice as either; ‘generalist’ (working to full scope of practice), ‘generalist with a special interest’ (extended scope of practice) or ‘specialist’ (reduced range, but extended scope of practice). Professional development activities were captured through participation in continuing education and professional qualifications. If practitioners considered themselves as holding a special interest in 3 or more fields of podiatry they were classified as generalist podiatrists, rather than specialists with three areas of interest. This was thought to reflect the intent of the question. Further questions were asked of those who considered themselves a specialist or held a special interest, about the area of special interest.

The area of special interest, or specialty, number of hours of continuing education spent on this area, membership of a special interest group, and tertiary qualification in the area were asked as in depth questions for those indicating a special interest or specialisation. Detailed data were obtained about the actual specific activities (treatments and patient assessment/management) that were performed in a clinical podiatry situation within the last week of practice. This important information was obtained in order to explore the association between actual and perceived scope of podiatry practice.

This study used basic descriptive statistical analysis with most data being categorical. Years of experience, and hours of working in a particular work setting, were recorded as ordinal data. The appropriate test of association between categorical variables, chi square tests using SPSS 19.0 for Windows (SPSS Inc., Chicago, IL, USA) was administered. Chi square tests can be administered when there are categories that are mutually exclusive, therefore data was arranged to reflect either being a specialist with a maximum of 2 areas of special interest or being a generalist (practicing full scope). Similarly in reviewing activities related to an area of special interest, people were classified dichotomously as either having the area of special interest (e.g. musculoskeletal) or no special interest in that area.

## Results

The total sample size was 218 responses, representing 13 % of the approx. 1600 population of member podiatrists who received the scope of practice survey through state based associations.

Thirty-four percent of respondents were male; This is a similar gender distribution to that reported by the Podiatry Board of Australia (PBA) in their December 2014 Podiatry Registrant Data Report on all registered podiatrists [11]. The modal group for years of experience in this study sample was 0–9 years. The PBA reports a modal age group of 26–30 years. Both population samples represent a substantially young profession. It would appear from the data (Table 1), that about half of responding podiatrists work full time (between 33 to 40 h per week) on average; there were however, a large group of people in part-time employment, most of these being female.

**Table 1:** Descriptive statistical results frequency analysis

Characteristics	Self - perceived generalist (*n* = 96)	Self - perceived specialist (*n* = 114)	Totals	Percentage	Analysis
Gender					
Female	70	68	138	66 %	
Male	26	46	72	34 %	ChiSq (df = 1, *P* = 0.044)
Missing			8		
Years experience					
0–9 years	24	50	74	34 %	
10–19 years	27	32	59	27 %	
20–29 years	25	23	48	22 %	
30–39 years	7	19	26	12 %	
40+ years	6	5	11	5 %	ChiSq (df = 2, *P* = 0.142)
Location					
Urban	64	61	128	58 %	
Rural and remote	18	18	38	17 %	ChiSq (df = 1, *P* = 0.899)
Missing			52	24 %	
What is your primary work setting?					
Private practice	82	81	163	75 %	
Public sector	11	31	42	19 %	
Education	0	3	3	1 %	
Missing			10	5 %	
Primary work environment					
Multipod	42	44	88	40 %	
Solo pod	35	33	71	32 %	
MDT	17	37	55	25 %	ChiSq (df = 3, *P* = 0.103)
Missing			4	2 %	
Work hours					
Part-time	50	51	111	51 %	
Full-time	35	62	97	44 %	ChiSq (df = 1,*P* = 0.190)
Missing			10	5 %	

The study participants were principally from private practice (75 %). From the survey group a majority of practitioners work in multiple podiatrist practices with up to a third working in solo practices. Multidisciplinary teams only make up a quarter of the surveyed group. Self -perception responses from survey participants were distributed between generalists (*n* = 96) generalists with a special interest (*n* = 107) and specialist (*n* = 15). Self-perception of specialist/one to two special interests status was compared with gender, years of experience, location, primary work environment and clinical practice.

For practitioners who declared a specialty or special area of interest further questions were asked of these subsets. There were two areas of special interest with very similar group sizes: Sports and biomechanics (*n* = 65) and Diabetes (*n* = 63). Analysis was performed for a relationship for categorical variables using Chi square tests, α level of significance (0.05), degrees of freedom (df) are as tabled. Comparisons were made within the biomechanics specialist group to find if there were activities that adequately described a podiatrist with a special interest in biomechanics.

There were many activities that are related to self -perception of biomechanics specialist see Table 2. The most significant activities were conveyed through Chi square analysis (df = 1, *P* = 0.0000) performed by those with a special interest in sport/biomechanics. These activities were: use of computer aided design and computer aided manufacture (CADCAM) orthoses, use of heel lifts, request for ultrasound, performing foot mobilisation, using a 3D scan technique in orthotic manufacture. Similarly there were activities that podiatrists performed that distinguished them as specialists in the diabetic foot, such as wound debridement, pathology for a wound swab and total contact cast see Table 3. The work environment and work setting were influential in perceptions of specialist status. See Table 4.

**Table 2:** Analysis of special interest area

	No special interest in sports/biomechanics (*n* = 152)	Special interst in sports/biomechanics (*n* = 65)		
Activity performed by practitioner	Number	Number	Total for each activity	Analysis
Use of CADCAM orthoses	18	24	42	*
Use of heel lift	71	51	122	*
Request ultrasound	54	41	95	*
Perform foot mobilisation	23	27	50	*
3D scan	15	20	35	*
Refer to orthopaedic surgeon	46	35	81	**
Customised non-cast orthoses	68	46	114	**
Gait assessment and report	85	53	138	**
Refer for x-ray	91	54	145	***
Custom orthoses	74	43	117	****
Totals	152	65		

Chi Square tests all had df = 1, *P = 0.000, **P = 0.001, ***P = 0.002, ****P = 0.025

**Table 3:** Analysis of special interest area. An anlaysis of ’Diabetes’ as an area of special interest

	No special interest in diabetes	Special interest in diabetes		
Activity performed by practitioner	Number (*n* = 151)	Number (*n* = 63)	Totals	Analysis
Wound debridement	92	56	214	*
Pathology swab	5	14	19	*
Ankle brachial index	29	24	53	**
X-ray	97	48	145	***
Totals	151	63		

Chi Square tests all had df = 1, *P = 0.000, **P = 0.002, ***P = 0.054

**Table 4:** Relationship of special interest to work. An exploration of two specific situations, primary work setting and primary work environment

Primary work setting	Private sector (*n* = 163)	Public sector (*n* = 42)	Totals	Analysis
Biomechanics special interest	61	4	65	Chi Sq (df = 2, *P* = 0.003)
Diabetes special interest	35	28	63	Chi Sq (df = 2, *P* = 0.000)
Podopaediatrics special interest	20	6	26	Chi Sq (df = 2, *P* = 0.690)
Primary work environment	Multi-podiatrists (*n* = 86)	Multi-disciplinary team (*n* = 54)	Solo podiatrist	Analysis
Biomechanics special interest (*n* = 65)	32	13	20	Chi Sq (df = 2, *P* = 0.217)
Diabetes special interst (*n* = 63)	14	28	21	Chi Sq (df = 2, *P* = 0.000)

Work environment with regards to sector was significant with biomechanics specialists’ predominately in private practice ChiSq (df = 2, *P* = 0.003). Podiatrists with a special interest in Diabetes were more aware of their special interest/specialisation when working in the public sector, ChiSq (df = 2, *P* = 0.000) and a multi-disciplinary team environment: ChiSq (df = 3, *P* = 0.000).

## Discussion

### Characteristics of a specialist podiatrist

This paper evaluates the perceptions and attributes of practitioners’ with a full scope of practice (generalist) compared with podiatrist who have a special interest or self perceived (specialist) scope of practice. Table 1 illustrates when practitioners are asked to categorise themselves to be either ‘generalist’ or ‘specialist/generalist with a special interest’ or ‘specialist’ podiatrist, male gender was identified as being the only factor which would predict perception of status (Chi square, df =1, P = 0.044). In this study the lesser proportion (34 %) of participants were male, however 64 % considered themselves to be specialists or working in an area of special interest. This may reflect more males being in the full-time workforce for longer periods. Exploring gender differences would be a good area for further research. Self -perception of specialist status was not explained by the variables: years of experience, location, working in rural versus urban environment, state worked in, or part-time/full-time work status.

Currently there is only one area of specialisation recognised by the Podiatry Board of Australia: podiatric surgery with the protected title of podiatric surgeon. A person must have worked as a generalist registered podiatrist for 2 years prior to pursuing an approved program of study. Fellowship of the Australian College of Podiatric Surgeons is gained through 3 years full time study. Alternatively the Doctor of Clinical Podiatry program is available through the University of Western Australia. All Australian University graduates from podiatry degrees (except unaccredited courses) now have sufficient qualification to meet the requirements of the Podiatry Board of Australia for endorsement for scheduled medicines.

As podiatric scope expands in Australia to recognise the specialty of podiatric surgery and endorsement for prescribing rights; it will be interesting to re-evaluate practitioners views on scope of practice in the future and whether this extended scope of practice is truly a new descriptor for future podiatrists scope of practice [12, 13]. The question arises: is measuring ‘specialisation or special interest’, simply a quasi marker for professional status [14]?

The only activity that adequately described a ‘specialist interest’ categorised podiatrist was the request for x-rays, not the similar activity of requesting diagnostic ultrasound. This is mostly explained by the two most shared areas of special interest: biomechanics/sports and diabetes/high risk foot both frequently requiring x-ray to assist diagnosis, but for different presentations or conditions.

### Analysis of specialisation area

Those participating clinicians who described themselves as a specialist or have a special interest in sports/biomechanics were characterised by being in the private sector and not by the work environment- of being in a multi-podiatrist practice– (see Table 4). There were also podiatric practise activities undertaken that characterised this area of special interest, such as three dimensional foot scan, Computer aided design- CADCAM orthoses, custom orthoses, customised non-cast orthoses, use of heel lift, performing foot mobilisation, request of ultrasound and x-ray and refer to orthopaedic surgeon. The development of skills in biomechanics appears to be part of working to full scope of practice for Australian podiatrists, however Kraus suggests this may be a diminishing scope of practice in young practitioners in the United States of America [15, 16]. The cultural context of health service delivery appears to influence pursuit of opportunity and rewards of specialisation.

Awareness of special interest in diabetes or the high-risk foot appears to be heightened by work setting in public sector and working in a multi-disciplinary team in Australia- (see Table 4). This awareness more closely resembles the work environment in the United Kingdom’s NHS and the development of specialisms [17]. The Australian experience of working in a multi-disciplinary team presents some likely challenges to professional boundaries. This is more likely to be the case in the public sector than private where roles around wound care and diabetes are defined by authorisations and work capacity [5, 18, 19].

Activities that were statistically significant for those with a special interest in diabetes/high risk foot at the *P* = 0.05 level for Chi-square tests were: wound debridement, pathology swab, total contact cast, taking of ankle-brachial index (ABI) and requests for x-ray. This raises the question as to whether these activities are within the scope of a generalist podiatrist and more frequent performing of these activities is associated with special area of interest or whether additional training is required to recognise an increased skill set.

### Future scope of practice in Australia

The future scope of practice in podiatry is dependent on external factors such as government legislation, funding for training, population ageing and demand for services, particularly with chronic disease and medical dominance of the health system. The Health workforce of the future according to the National Health Workforce Innovation and Reform Strategic Framework 2011–2015 should:

“Develop an adaptable health workforce equipped with the requisite competencies and support that provides team-based and collaborative models of care” [20].

This model is contrary to traditional status gaining roles of specialisation which we have shown are still pursued within the podiatry profession in Australia. Will the proposed future model of collaborative care facilitate professional satisfaction and will it be embraced by podiatry, which is principally managed by private practitioners?

Internal drivers will be determined from within the professional body and are subject to external influences. The future will involve promoting the status of the profession through recognition by role extension and task substitution to gain the full scope of practice. Future difficult negotiations and interaction with other health professionals in an inter-professional manner will determine boundary negotiation [14, 21–23].

### Study limitation

This study was originally designed as an industry lead review and thus questions were formatted in a particular way to elicit these responses. This has produced some recall and information bias, with some small sample groups, which were too small for Chi-square analysis. In comparisons between ‘generalist’ and ‘specialist’, groups were combined into ‘specialist’ if they either called themselves ‘specialist’ or had a special interest in one or two areas, thus making the groups more dichotomous. The population sample was limited by distribution of the survey, with an uneven uptake by only 13 % of the profession. The sample size was only 218 interested respondents and is a clear limitation to the study, which was undertaken through voluntary responses of members of the APodC. This still remains a timely report on the current state of the profession at a critical time of change with recent introduction of specialisation and national registration.

## Conclusion

It is clear that the Australian health workforce is undergoing change, responding to health consumer population and demographic forces and government priorities. Regulation of health professions in Australia has fundamentally shifted from a state based to nationalised system and one of protectionism of scope to protection of title only.

Podiatry Board of Australia has acknowledged the need for recognition of podiatric surgeon as a specialist category, yet many other practitioners relate to the term specialist or have a special interest within podiatry. It is suggested that recognition of specialisation is different for regularity authorities to practicing podiatric clinicians. Regularity authorities recognising public safety as a key determinant of need for specialisation, however practicing clinicians awareness of specialistisation is related to gender, work environment and setting and is associated with performing of a number of treatment related activities. This is in direct contrast to government agencies such as Health Workforce Australia who encourage fostering of generalist skills, and delivery of care through collaboration and innovation to meet the health care needs of all Australians.

Health workforce reform in Australia is driven principally by factors external to the profession that affects the entire health workforce. The podiatry profession in Australia is different to most other health professions in being principally private practice, not working in multi-disciplinary teams, being young, female biased and pursuing specialist areas of interest.

## Availability of supporting data

Original data are available on request to the corresponding author.

## Additional file

Additional file 1:
**Original scope of practice electronic survey.** (DOCX 39 kb)

## Abbreviations

AHPRA: Australian health practitioner regulation agency
AHWI: Australian health workforce institute
ANZPAC: Australia and New Zealand accreditation council
COAG: Councils of Australian governments
HWA: Health workforce Australia
NHS: National health service
NRAS: National registration and accreditation scheme
PBA: Podiatrists board of Australia
