# Supplementary material for: Specialisation versus special interest - the Australian podiatry experience
Source: J Foot Ankle Res. 2015 Dec 3;8:69. doi: 10.1186/s13047-015-0127-0 (PMC4669669; doi:10.1186/s13047-015-0127-0)
Supplement: Additional file 1: — Original scope of practice electronic survey. (DOCX 39 kb) [file 13047_2015_127_MOESM1_ESM.docx]

Exit this survey


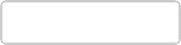


**100%**


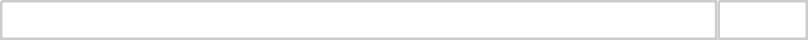


# What is your gender?


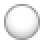
 Female
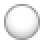
 Male

# How many years of experience do you have as a podiatrist?


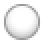
 0-4


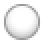
 5-9


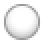
 10-14


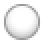
 15-19


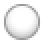
 20-24


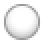
 25-29


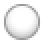
 30-34


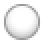
 35-39


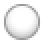
 40+ years

# In which Australian state do you work?


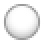
 A.C.T.


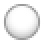
 New South Wales
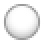
 Northern Territory
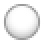
 Queensland


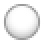
 South Australia
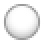
 Tasmania


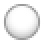
 Victoria


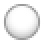
 Western Australia

# What is your work setting? Average hrs per week in Primary clinical work setting

0-8 hrs
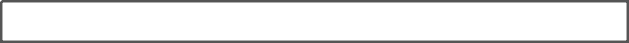
 9-16hrs
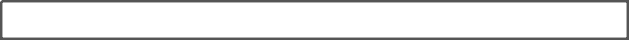
 17-24 hrs
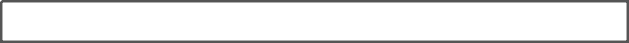
 25-32hrs
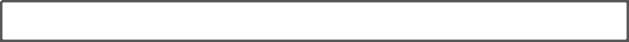
 33-40 hrs


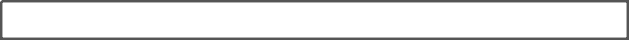


41< hrs


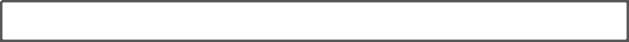


# What is your primary work setting?

hospital urban rural remote public
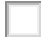

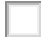

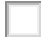

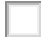
 private
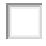

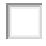

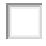

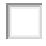
 community
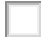

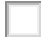

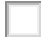

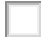
 education
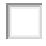

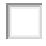

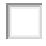

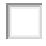


# What is your primary work environment?


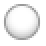
 Multiple podiatry practice
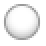
 Multi-disciplinary team
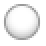
 Sole practitioner

# What is your work setting? Average hrs per week in Secondary clinical work setting

0-8 hrs
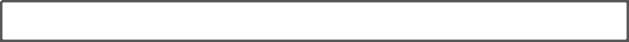
 9-16hrs
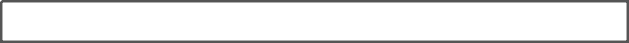
 17-24 hrs
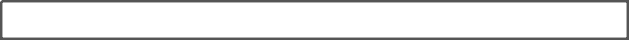
 25-32hrs
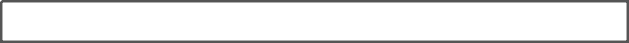
 33-40 hrs
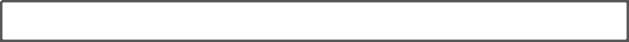


41< hrs
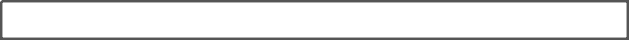


# What is your secondary work setting?

hospital urban rural remote public
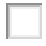

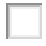

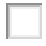

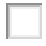
 private
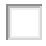

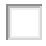

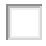

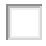
 community
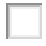

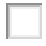

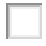

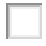
 education
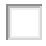

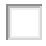

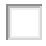

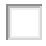


# What is your secondary work environment?


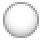
 Multiple podiatry practice
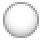
 Multi-disciplinary team


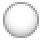


Sole practitioner

# What type of podiatrist do you consider yourself?


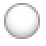
 Generalist podiatrist


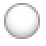
 Generalist with a special interest
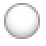
 Specialist podiatrist

# If special interest or specialist what specialty?


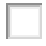
 High risk/diabetes
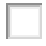
 Paediatrics


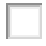
 Sports/biomechanics Other (please specify)


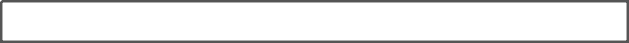


# How many hours of continuing education would you have done in this area of specialty in the last 12 months?

1- 8
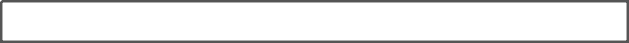


9-16
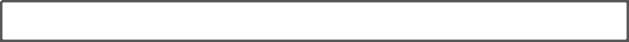


17-24
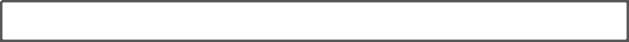


25-32,
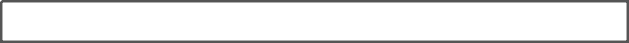


33-40
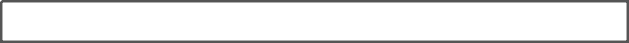


41< hours
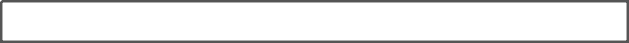


# Are you a member of a special interest group in this area of specialty?


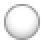
 Yes
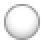
 No

# If you are a member of a special interest group: What is the name of the group?


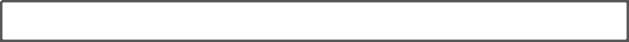


1. **What is the highest level of professional qualification you have completed?**


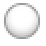
 Diploma,
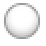
 Bachelor,


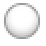
 Double degree,
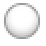
 Honours,


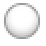
 Grad Cert,
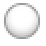
 Grad Dip,


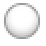


Masters,

PhD/DPM


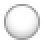


Other (please specify)


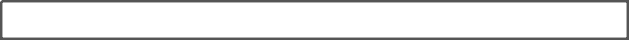


# If you hold a higher degree, what Major or research topic did you pursue?


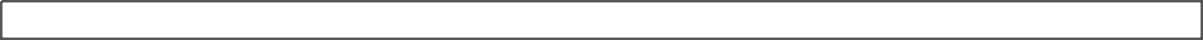


1. **Which of the following activities did you perform in treating patients in the last week?**


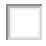
 Nail care


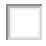
 Laser treatment for fungal nail
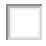
 Corn and callus debridement

Wound debridement and dressing Padding and Strapping

Stretching/strengthening exercises Massage

Mobilisation

Footwear assessment and advice Gait assessment with report

Plaster cast of feet 3D scan

Custom cast orthoses CAD CAM orthoses

Customised non-cast orthoses / insoles Shoe padding

Off the shelf orthoses/insoles Heel lift

Dry needling/acupuncture Prolotherapy

Platelet rich plasma injections

Indirect/ direct refer to vascular specialist? Indirect/ direct refer to orthopaedic surgeon? Indirect/direct refer to podiatric surgeon?

Ingrown nail surgery Total contact casting

A.B.I.

Refer for ultrasound of foot/ankle Refer for CT of foot/ankle

Refer for MRI of foot/ankle Request pathology for nail

Request pathology for blood tests Request pathology for swab

Request pathology for skin biopsy Use of 1% Lignocaine

Use of LA other than 1% Lignocaine Prescription for endorsed S4 drugs

Other (please specify)

Done

Powered by **SurveyMonkey**

Check out our sample surveys and create your own now!
